# FR4-Based Electromagnetic Scanning Micromirror Integrated with Angle Sensor

**DOI:** 10.3390/mi9050214

**Published:** 2018-05-02

**Authors:** Hongjie Lei, Quan Wen, Fan Yu, Ying Zhou, Zhiyu Wen

**Affiliations:** 1 Microsystem Research Center, College of Optoelectronic Engineering, Chongqing University, Chongqing 400044, China; Yu_Fan@cqu.edu.cn (F.Y.); yzhou@cqu.edu.cn (Y.Z.); wzy@cqu.edu.cn (Z.W.); 2 Key Laboratory of Fundamental Science of Micro/Nano-Device and System Technology, Chongqing University, Chongqing 400044, China; 3 Fraunhofer Institute for Electronic Nano Systems (ENAS), 09131 Chemnitz, Germany

**Keywords:** scanning micromirror, electromagnetic actuator, angle sensor, flame retardant 4 (FR4)

## Abstract

This paper presents a flame retardant 4 (FR4)-based electromagnetic scanning micromirror, which aims to overcome the limitations of conventional microelectromechanical systems (MEMS) micromirrors for the large-aperture and low-frequency scanning applications. This micromirror is fabricated through a commercial printed circuit board (PCB) technology at a low cost and with a short process cycle, before an aluminum-coated silicon mirror plate with a large aperture is bonded on the FR4 platform to provide a high surface quality. In particular, an electromagnetic angle sensor is integrated to monitor the motion of the micromirror in real time. A prototype has been assembled and tested. The results show that the micromirror can reach the optical scan angle of 11.2° with a low driving voltage of only 425 mV at resonance (361.8 Hz). At the same time, the signal of the integrated angle sensor also shows good signal-to-noise ratio, linearity and sensitivity. Finally, the reliability of the FR4 based micro-mirror has been tested. The prototype successfully passes both shock and vibration tests. Furthermore, the results of the long-term mechanical cycling test (50 million cycles) suggest that the maximum variations of resonant frequency and scan angle are less than 0.3% and 6%, respectively. Therefore, this simple and robust micromirror has great potential in being useful in a number of optical microsystems, especially when large-aperture or low-frequency is required.

## 1. Introduction

The scanning micromirror is a promising component for wide applications, such as projection displays [1], barcode readers [2], micro-spectrometers [3,4] and biomedical imaging [5]. Currently, most of the scanning micromirrors are developed and fabricated using the microelectromechanical systems (MEMS) technology and can be driven by different actuation mechanisms, such as electrostatic [6,7], electrothermal [3,8], electromagnetic [4,9,10] and piezoelectric mechanisms [11,12]. The silicon MEMS micromirror shows exceptional properties that are suitable for high frequency scanning applications due to its small size, low power consumption and fast speed [13]. However, the low-frequency (in the order of a few hundred Hz) silicon MEMS micromirror is fragile and cannot survive the environmental shocks and vibrations due to the brittleness of silicon [14]. Moreover, the fatigue strength of silicon decreases when the size of MEMS structure increases [15], thus dramatically limiting the aperture of the mirror plate.

Currently, a low-frequency scanning and large-aperture micromirror is required for a broad spectrum of applications, such as micro-spectrometers [4], laser projection [16,17,18], fluorescence microscopes [19] and so on. It has been a continuous and ongoing task to find a proper alternative in order to meet the vast application demand. Some groups have proposed the use of metal instead of silicon as the substrate material in the scanning micromirror devices [19,20,21,22]. The metal-based micromirror possesses stronger robustness due to the ductile properties of the metal compared with silicon. However, the metal substrate usually needs an additional separation process to form actuators, inevitably increasing the process complexity and the production cost. Moreover, the surface quality of the metal-based micromirror is inferior to that of the silicon micromirror. Another inexpensive yet highly suitable candidate is flame retardant4 (FR4) [17,18,23], which is inherently a soft material with a low Young’s modulus of about 20 Mpa. It is the most widely used material for printed circuit boards (PCB) due to its good electrical, mechanical and thermal properties. Thus, a robust scanning micromirror can be quickly fabricated using FR4 as the substrate through the commercially available and low-cost PCB fabrication process.

In this paper, a FR4-based electromagnetic scanning micromirror is proposed in order to overcome the limitations of the conventional MEMS micromirrors for large-aperture and low-frequency scanning applications. The copper coils for the actuation are printed on the bottom layer of a thin FR4 platform. An aluminum-coated silicon mirror plate with a large aperture (11.7 mm × 10.3 mm) is bonded on the FR4 platform to provide a high surface quality. Particularly, an electromagnetic angle sensor with double-layer sensing coil is integrated on the same FR4 platform without the requirement of an additional process. The angle sensor can monitor the deflection angle in real time, which is very useful for a micromirror as it forms precise closed-loop control [24,25]. The innovation lies in that both the actuator and angle sensor are simultaneously fabricated on a FR4 board using a low-cost PCB process instead of the expensive Si-based MEMS process. Furthermore, the device is fully packaged and tested to demonstrate its great performance in terms of driving, sensing and reliability. The rest of this paper is organized as follows. The design and theory of the electromagnetic micromirror integrated with the angle sensor is introduced in Section 2. After this, the tests and corresponding results of actuation, sensing, response and reliability are described in Section 3. Finally, a brief conclusion is given in Section 4.

## 2. Design and Theory 

**Figure 1:**
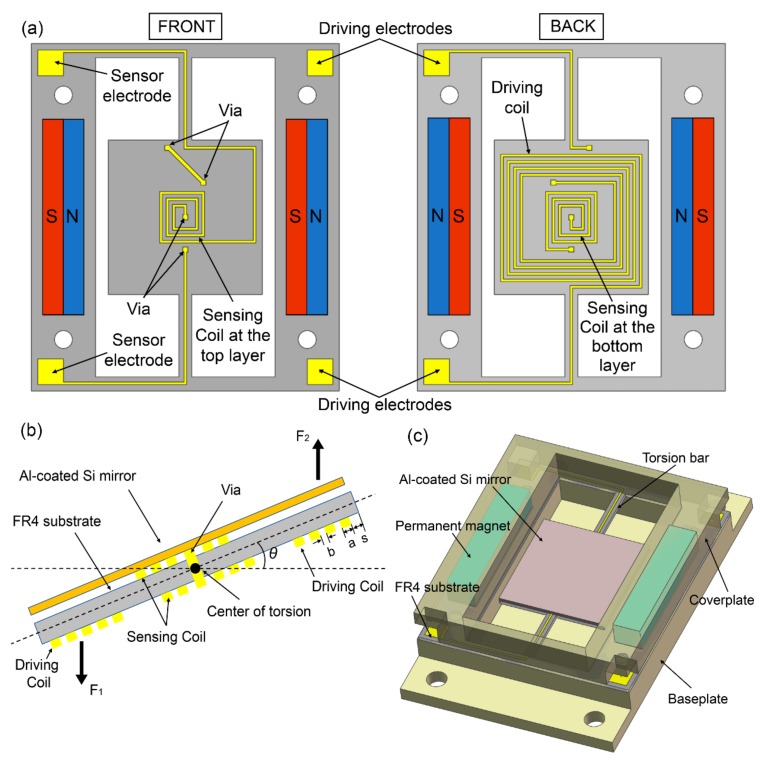
(**a**) Layout of the flame retardant 4 (FR4) platform with double-layer copper coils; (**b**) Electromagnetic actuation and sensing of the FR4 platform with the attached Al-coated Si mirror plate; and (**c**) Schematic drawing of the assembled scanning micromirror.

The proposed FR4-based electromagnetic scanning micromirror is shown in Figure 1. It includes a 400-μm-thick FR4 platform, a 500-μm-thick silicon mirror plate on it and a pair of permanent magnets. The layout of the FR4 platform is shown in Figure 1a. Both the outer single-layer driving coil (on the bottom layer) and the inner double-layer sensing coil are simultaneously integrated into this platform. Furthermore, the top and bottom layers for sensing coil are connected with vias. The 12 mm × 12 mm central platform is anchored to the frame by a pair of torsion bars. The length and width of the torsion bars are 11 mm and 1 mm, respectively. Two permanent magnets are assembled in parallel, generating a magnetic field that is mainly parallel to the mirror. When the driving coil is energized, a Lorentz force is generated to exert a net torque about the torsion axis. Consequently, the platform with the mirror plate is actuated to tilt, which is shown in Figure 1b. At the same time, the sensing coil induces an electromotive force in this platform. Therefore, the deflection angle can be monitored in real time. Figure 1c shows the schematic drawing of the assembled scanning micromirror. Both the FR4 platform and permanent magnets are sandwiched between the baseplate and coverplate.

The motion of the scanning micromirror can be approximated as a forced oscillating system and thus, the corresponding dynamical equation can be expressed as:

(1) $$J_{m}\ddot{\theta }+C\dot{\theta }+K\theta =T$$

where *J_m_*, *C*, *K*, *T* and *θ* present the moment of inertia of the platform with the mirror plate around the torsion axis, the damping coefficient, the torsional stiffness of the torsion bars, the net torque and the mechanical half deflection angle, respectively. As the driving signal is sinusoidal with the resonant frequency *f*, by solving Equation (1), the mechanical half deflection angle can be obtained as:

(2) $$\theta =\frac{T}{K}Q$$

where *Q* = 1/2*ξ* is the quality factor and *ξ* represents the damping ratio.

When the mechanical half deflection angle is small (cos *θ* ≈ 1), the net torque can be approximately described as:

(3) $$T=iBM_{d}$$

where

(4) $$\begin{array}{l} M_{d}=\sum_{n=1}^{N_{d}}\left(\frac{w-2s-b}{2}-\left(n-1\right)\left(a+b\right)\right)\times \left(\left(2l-4s-3a-5b\right)-4\left(n-1\right)\left(a+b\right)\right)\\ -\left(\frac{w-2s-b}{2}-\left(N_{d}-1\right)\left(a+b\right)\right)\times \left(\left(l-2s-2a-3b\right)-2\left(N_{d}-1\right)\left(a+b\right)\right) \end{array}$$

where *i* is the driving current; *B* is the magnetic flux density produced by the magnets; *M_d_* is the total area of all the driving coils; *l* and *w* are the length and width of the FR4 platform, respectively; *N_d_* is the number of the driving coil turns; *a* is the spacing of adjacent coils; *b* is the width of coil; and *s* is the width of the exterior border zone of the platform. According to Equations (2)–(4), it is easy to predict the micromirror scan angle.

At the same time, the electromotive force induced in the sensing coil can be approximately described as:

(5) $$\varepsilon_{s}=BM_{s}\dot{\theta }$$

where

(6) $$\begin{array}{l} M_{s}=2\sum_{n=1}^{N_{s}}\left(\left(a+\frac{3}{2}b\right)+\left(n-1\right)\left(a+b\right)\right)\times \left(\left(5a+7b\right)+4\left(n-1\right)\left(a+b\right)\right)\\ -\left(\left(a+\frac{3}{2}b\right)+\left(N_{s}-1\right)\left(a+b\right)\right)\times \left(\left(3a+4b\right)+2\left(N_{s}-1\right)\left(a+b\right)\right)\\ +\left(\frac{w}{2}-s\right)\times \left(\left(a+\frac{3}{2}b\right)+\left(N_{s}-1\right)\left(a+b\right)+\left(\frac{l}{2}-s\right)\right) \end{array}$$

where *M_s_* is the total area of the all the sensing coils; and *N_s_* is the number of the sensing coil turns. All the aforementioned parameters of the FR4 platform are listed in Table 1. The mechanical half deflection angle *θ* can be described by means of the maximum mechanical half deflection angle *θ*_0_ and the phase shift *φ* with respect to the driving voltage as follows:

(7) $$\theta =\theta_{0}\sin\left(2\pi ft+\varphi \right)$$

By substituting Equation (7) to Equation (5), the electromotive force induced in the sensing coil can be expressed as:

(8) $$\varepsilon_{s}=2\pi fBM_{s}\theta_{0}\cos\left(2\pi ft+\varphi \right)$$

Therefore, the scanning angle can be attained in real time through the voltage output of the angle sensor. Moreover, the amplitude of the induced electromotive force is proportional to the maximum deflection angle of the micromirror.

## 3. Test Results of Prototype

### 3.1. Optical Scan Angle

**Figure 2:**
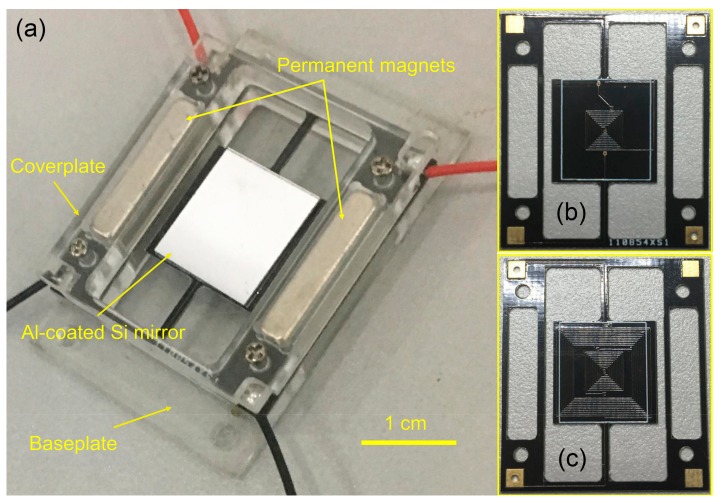
(**a**) The photograph of the prototype of FR4-based electromagnetic scanning micromirror with a simple plexiglass package; (**b**) Front-side of the FR4 platform integrated with copper coils for sensing; and (**c**) Back-side of the FR4 platform integrated with copper coils for driving and sensing.

**Figure 3:**
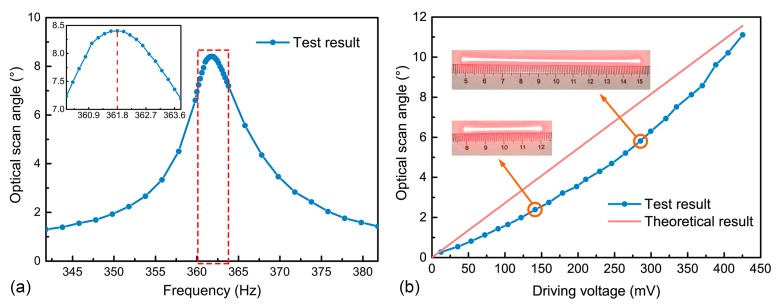
Optical scan angle test of the FR4-based scanning micromirror: (**a**) Optical scan angle versus frequency with fixed driving voltage and the resistance of the driving coil being 3.7 Ω; and (**b**) Optical scan angle versus driving voltage at its resonant frequency.

Figure 2 shows the prototype of the FR4-based electromagnetic scanning micromirror with a simple plexiglass package. The performance of the device is tested by shooting a laser spot on the mirror plate and measuring the length of the projected laser line. After this, the scan angle can be calculated according to the length of the projected laser line and the distance between the projected screen and the mirror plate. A sinusoidal voltage is applied to the driving electrodes to actuate the micromirror. Therefore, it is convenient to find the resonant frequency and test the actuation performance by adjusting the driving signal frequency and driving voltage amplitude, respectively. Figure 3a shows the results of the micromirror optical scan angle with changes in the frequency. According to the test results, its resonant frequency is 361.8 Hz when it reaches the maximum scan angle and the 3-dB bandwidth is 6.16 Hz. Hence, the quality factor of *Q* = 59 and damping ratio of *ξ* = 0.0085 can be obtained by the half-power bandwidth method. Figure 3b shows the relationship between the optical scan angle and the driving voltage amplitude when the driving frequency is fixed at its resonant frequency of 361.8 Hz. The optical scan angle increases with an increase in the driving voltage and can reach the maximum value of 11.2° at 425 mV. This result is very close to the theoretical value. The slightly non-linear nature of the test curve could be caused by the non-linear spring effect of the torsion bars [26]. The measured resistances of the driving coil and sensing coil are 3.7 Ω and 2.4 Ω, respectively.

### 3.2. Angle Sensor

**Figure 4:**
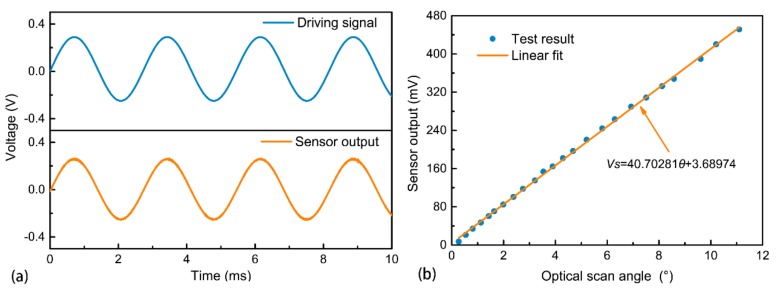
Measurement of the integrated angle sensor: (**a**) Time dependence of the driving signal and the sensor output signal; and (**b**) Relation between the sensor output signal and the optical scan angle.

The output signal of the angle sensor is amplified by a simple amplifier with a gain of 400. After this, it can be measured using an oscilloscope. As seen in Figure 4a, the sensor output shows a good signal-to-noise ratio and is approximately in phase with the driving signal. This result indicates the good feasibility of using the sensing coil as an angle sensor. The value of the signal-to-noise ratio can be obtained as 43 dB through a fast Fourier transform for the sensor output in the time domain. Furthermore, the phase relation can be predicted theoretically. According to Equation (5), the electromotive force induced in the sensing coil is in phase with the angular velocity of the micromirror, while the angular velocity is in phase with the driving voltage when the micromirror is actuated at its resonant frequency. Consequently, the electromotive force is in phase with the driving voltage, which means that the resonance can be tracked by monitoring the phase difference between the driving and sensing signals.

Figure 4b plots the relationship between the sensor output voltage and the micromirror optical scan angle at the resonance point. The sensor signal is proportional to the optical scan angle of the micromirror. We determined a significant linear relation with a correlation coefficient of *r* = 0.99963. This is consistent with the theoretical relation determined by Equation (8). The sensitivity of the angle sensor can be determined as 40.70281 mV/°, which is enough for feedback control [12]. According to Equation (8), the theoretical value of sensitivity is 44.2131 mV/°. The difference between the theoretical and experimental values could be attributed to the fabrication error and coupling influence between the driving and sensing coils.

### 3.3. Response Test

**Figure 5:**
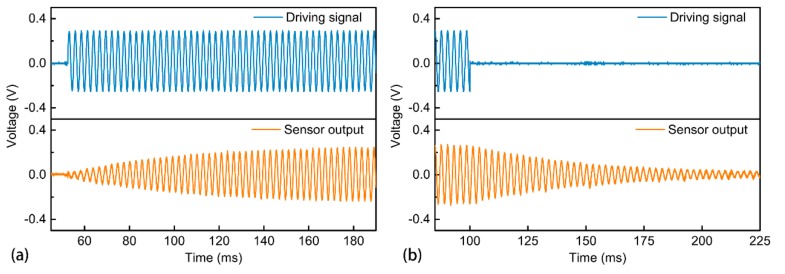
Test of: (**a**) the transient response and (**b**) damping ratio of the micromirror through the integrated angle sensor.

Due to the integration of the angle sensor, it is very convenient to test the dynamic response of the micromirror through the sensor signal without the need of an external experimental setup. A sinusoidal driving signal of 361.8 Hz, which is the resonant frequency, is applied. The results are shown in Figure 5. According to the test result in Figure 5a, it takes about 120 ms for the micromirror to reach stable oscillation. This short time will not cause any noticeable unstable scanning delay. Furthermore, the damping ratio can be obtained by the free vibration decay method shown in Figure 5b, with the corresponding value being 0.0081. The result obtained from the angle sensor is very close to the aforementioned value obtained from the micromirror scanning. This small error can be attributed to the measuring error and the difference between the two test methods of the damping ratio. Therefore, the accuracy of the sensor signal is further verified by the test result.

### 3.4. Reliability of Micromirror

The mechanical shock, vibration, and long-term cycling tests are all performed to evaluate the reliability of FR4-based electromagnetic scanning micromirror. In the mechanical shock, the prototype is tested in three mutually perpendicular axes. The amplitude of the shock is 1000 g with a duration of 1 ms. The results show that the prototype passes the shock test successfully. 

After this, the vibration test is carried out by mounting the prototype on the vibration machine. The amplitude of the vibration is 20 g with the sweeping frequency from 20 Hz to 1000 Hz and back in 20 min. It also passes the vibration test without any failure in the three mutually perpendicular axes.

**Figure 6:**
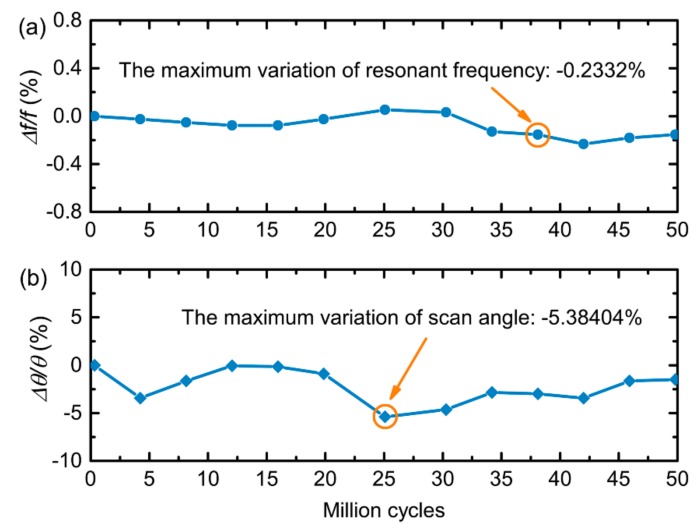
Long-term cycling test on the prototype by keeping it in resonance for nearly 50 million cycles, which shows the variation of: (**a**) resonant frequency and (**b**) scan angle in the test.

Finally, we perform a long-term cycling test on the prototype by keeping it in resonance for nearly 50 million cycles. Figure 6 plots the variation of the resonant frequency and scan angle. The maximum variations of both parameters are less than 0.3% and 6%, respectively, which demonstrates its great reliability and resistance against the stress and heat of FR4. The minor changes can be explained by the changing temperature and humidity in the laboratory environment during the nearly 40-hour test period [19]. Furthermore, the angle variation can be reduced or even eliminated by using the integrated angle sensor to form a precise close-loop feedback control system.

## 4. Conclusions

In summary, a prototype of FR4-based electromagnetic scanning micromirror integrated with an angle sensor is presented in this paper. It is designed and fabricated based on a commercial PCB technology for a short time period at a low cost. The device has a large aperture (11.7 mm × 10.3 mm) with high surface quality and low frequency, which is difficult to achieve with the conventional silicon MEMS. The test results show that the optical scan angle can reach 11.2° under a low driving voltage of only 425 mV at the resonant frequency of 361.8 Hz. Additionally, the signal of the integrated angle sensor is confirmed to be proportional to the optical scan angle of the micromirror. It has good signal-to-noise ratio, linearity and sensitivity. Thus, it can be used for the real-time angle monitoring and precise close-loop control. Finally, the reliability tests, including shock, vibration and long-term cycling, are carried out. The results show that the prototype possesses high reliability. Therefore, this simple and robust micromirror holds promise for a number of optical microsystem applications that require low-frequency scanning. In the future, the performance of the actuation and sensing can be further improved by employing a multilayer PCB process to increase the number of coil turns and some simple electronics can be even integrated on the same FR4 platform.

## Figures and Tables

**Table 1:** Parameters of the flame retardant 4 (FR4) platform integrated with driving and sensing coils.

Parameters	*l*	*w*	*N_d_*	*N_s_*	*a*	*b*	*s*
Value	12 mm	12 mm	11	9	0.1 mm	0.1 mm	0.5 mm
